# A test of oscillation in the human secondary sex ratio

**DOI:** 10.1093/emph/eoaa012

**Published:** 2020-04-21

**Authors:** Ralph Catalano, Joan A Casey, Tim A Bruckner

**Affiliations:** 1 School of Public Health, University of California, Berkeley, Berkeley, CA, USA; 2 Environmental Health Sciences, Columbia University, New York, NY, USA; 3 Program in Public Health, University of California, Irvine, Irvine, CA, USA

**Keywords:** oscillation, secondary sex ratio, Hamilton, cohort longevity

## Abstract

**Background and objectives:**

The sex ratio of human birth cohorts predicts the health and longevity of their members. Most literature invokes natural selection in support of the argument that heritable tendencies to produce male or female offspring induce oscillation in the sex ratio and its sequelae. Tests of the argument remain exceedingly rare because they require vital statistics describing many generations of a population both unaffected by migration and exposed to an exogenous stressor virulent enough to change the sex ratio at birth. We contribute to the literature by using time-series modeling to detect oscillation in the best data currently available for such a test.

**Methodology:**

We apply rigorous time-series methods to data describing Sweden from 1751 through 1830, a period when the population not only aged in place without migration, but also exhibited the effects of an Icelandic volcanic eruption including a historically low secondary sex ratio. That very low sex ratio should have induced oscillation if heritable mechanisms appear in humans.

**Results:**

We detected oscillation in the ratio but not that predicted by heritable tendencies to produce males or females. We found peak-to-trough oscillation at 14 rather than the approximately 32 years expected from the heritable tendencies argument.

**Conclusions and implications:**

Our findings suggest that mechanisms other than perturbation of heritable tendencies to produce males or females induce oscillation in the human secondary sex ratio. These other mechanisms may include reproductive suppression and selection *in utero*.

**LAY SUMMARY:**

The male to female ratio in human birth cohorts predicts longevity but its variation over time remains unexplained. We test the long-held theory that the ratio oscillates due to heritable tendencies to produce males or females. We find oscillation, but it appears due to social processes rather than heritable mechanisms.

## INTRODUCTION

Consistent with evolutionary theory [1], the health of human populations reportedly affects the ratio of males to females in birth cohorts (i.e. the secondary sex ratio) [2]. That ratio, in turn, affects the health of birth cohorts over the life course [3–5]. Males from cohorts with unusually low secondary sex ratios, for example, show a lower risk of fetal growth restriction [6], fewer birth defects [7, 8] and greater than expected cognitive function as children [5]. Males in higher sex ratio cohorts show greater mortality before adulthood [9] as well as diminished overall lifespan [3]. In light of these reports, understanding temporal variation in the sex ratio at birth would seem an important topic for research at the intersection of evolutionary theory, medicine and public health. 

Influential but rarely tested theory predicts that the ratio will exhibit oscillation induced by endogenous mechanisms as well as episodes of damping oscillation caused by exogenous shocks. We use the best available data to test these predictions.

Düsing and Fisher provided the seminal explanation of temporal variation in the secondary sex ratio [10–12]. Their argument assumed that individuals inherit the tendency to produce either male or female children and that these tendencies affect reproductive fitness depending on the sex ratio of the reproducing population. Hamilton later consolidated and simplified these arguments in his widely cited and highly influential summary of the Düsing/Fisher argument [13]. He wrote:

Suppose male births are less common than female.
A newborn male then has better mating prospects than a newborn female, and therefore can expect to have more offspring.
Therefore, parents genetically disposed to produce males tend to have more than average numbers of grandchildren born to them.
Therefore, the genes for male-producing tendencies spread, and male births become more common.
As the 1:1 sex ratio is approached, the advantage associated with producing males dies away.
The same reasoning holds if females are substituted for males throughout.

Hamilton’s summary implies that changes in the secondary sex ratio serve as both the cause and effect of the homeostasis suggested by Düsing and Fisher—a biased secondary sex ratio at time *t* produces an opposite, though diminished, bias at time *t + n*. Under Hamilton’s logic, *n* equals the age at which the cohort born at *t* reaches peak fertility.

Hamilton did not speculate on what might cause a biased secondary sex ratio in humans that would, in turn, induce oscillation. The assumption of natural selection working in combination with a genetic disposition to produce one or the other sex implies, however, at least two causes. First, a biased sex ratio could emerge from the stochastic nature of conception in the population. The stochastic draw of parents with differing genetic propensities to produce sons or daughters implies a normal distribution of, for example, annual sex ratios around an expected value close to one. Outlying deviates drawn from this distribution presumably trigger mechanisms that return ratios to values closer to expected. Secondary sex ratios would not, therefore, appear independent of each other over time but rather exhibit autocorrelation. Hamilton’s summary predicts that this endogenous autocorrelation induces oscillation peculiar to each birth cohort and its progeny over several generations. The time between peak and trough in this oscillation would equal the mean age at which birth cohorts reach greatest fertility and could, therefore, vary over generations as that age shifts. Although statistical analyses can, as described below, detect such endogenous oscillation, ‘seeing’ the pattern in a time-series plot of, for example, the annual secondary sex ratios for a society would prove difficult given the contribution of several parental birth cohorts to the secondary sex ratio in any year.

Biased secondary sex ratios in humans also likely arise from exogenous shocks, such as natural disasters, societal disruption and extreme weather that stress entire populations [14–16]. As argued by James, the human stress response likely reduces the sex ratio at conception (i.e. the primary sex ratio) by altering, in both males and females, the hormonal cascades that presumably influence the sex of offspring [17, 18]. Exogenous stressors may also lower the secondary sex ratios by affecting which fetuses survive gestation [19]. Most human conceptions do not produce a live birth [20]. While fetal loss before clinical recognition of pregnancy remains difficult to observe, it appears to select against chromosomally and genetically abnormal fetuses and, for reasons not understood, more against what would have been female than male infants [21]. Studies following pregnancies after clinical recognition (i.e. approximately 10th week of gestation) have shown that survivors to birth have fewer chromosomal and genetic abnormalities and include fewer small for gestational age, but otherwise ‘normal’, males than the population at the start of observation [22–24]. The literature has intuitively characterized this distillation of conception cohorts *via* fetal loss as ‘natural selection *in utero*’ because survivors to birth appear more reproductively fit than the starting population [19, 25].

Selection *in utero* against small male fetuses would seem adaptive for several reasons including that mothers of small sons have fewer grandchildren than other mothers particularly when those sons are born into environments that most threaten infant survival [26, 27]. This relatively low fitness arises, in part, from high death rates among young males. Male infants more likely die than any other sex by age group through reproductive age in every society and virtually every year for which we have dependable vital statistics [28].

Estimating the depth of selection *in utero* in conception cohorts remains difficult because clinicians rarely detect failure to implant or early fetal loss in populations not receiving fertility treatment. Researchers, therefore, often use the secondary sex ratio as a ‘tracer’ of selection *in utero* under the assumption that the ratio will appear unexpectedly low when selection has been unusually deep [19]. Published tests of the hypothesis that environmental threats to infant survival or maternal resources induce selection *in utero* often report lower sex ratios in exposed populations [29–31].

Hamilton’s summary implies that birth cohorts with outlying sex ratios caused by exogenous shocks to the population should trigger oscillation. Unlike the endogenous oscillation described earlier, however, exogenously induced oscillation can appear extreme and visually detectable. Yule referred to such oscillation as ‘superimposed’ because it appears over and above the endogenous oscillation caused, in the case of the human secondary sex ratio, by the stochastic nature of conception in a population [32].

Scholars have questioned, on theoretical grounds, whether Hamilton’s highly influential description of oscillating sex ratios applies to humans. Frank [33], for example, notes that the argument does not consider post-birth investments as well as life-history tradeoffs between current sex ratio and future reproduction in humans. Cockburn *et al.* [34] further note that the social structure of human societies, which include complicated interactions across overlapping generations, may violate the conditions assumed by Hamilton’s argument.

The literature includes attempts to test Fisher’s assumption of a heritable tendency to produce males or females [35–41]. That work uses various methods and sampling strategies but fails to agree on whether such a tendency appears in humans.

Although the empirical literature includes several tests of the hypothesis that the sex ratio of reproductive aged humans (i.e. the operational sex ratio) at time *t* predicts the secondary sex ratio at *t* [42–47], we know of only one offering evidence related to oscillation. Song [48] found support for the hypothesis that women born in China from 1962 through 1964, when famine depressed the secondary sex ratio, would, when reaching reproductive age, produce more males than expected from births to other women.

Song did not offer his findings as evidence for Fisher’s and Hamilton’s argument that the sex ratio will oscillate due to a heritable tendency to produce offspring of one or the other sex. In fact, he did not invoke such a tendency when introducing his hypothesis or when discussing his findings. He, rather, interpreted his findings as support for the argument that early childhood experiences of females somehow program the sex of their offspring [46, 49]. As Song acknowledged, moreover, selective abortion affected his results. His estimates, in fact, rejected the null only after adjusting sex ratios for self-reported abortions. Reports published since his work [50] provide, however, evidence of considerable illegal, and unlikely acknowledged, female-specific abortions in the years when women born from 1963 through 1964 would have contributed significantly to annual birth cohorts.

Other empirical work [41] has attempted to test the assumption of a heritable tendency to produce male or female offspring. This work reports that the sex of an individual’s offspring does not predict the sex of the offspring of the individual’s siblings. That work, however, did not estimate the association between the sex of an individual’s offspring and the sex of that individuals grandchildren.

We contribute to the literature by using data of unusually high quality to test two hypotheses implied by Fisher’s and Hamilton’s arguments. First, annual human secondary sex ratios exhibit autocorrelation in the form of oscillation with time between peak and trough equal to age at which women and men reach peak fertility. Second, extreme sex ratios associated with an exogenous shock predict opposite, though damping, outliers at a lag equal to the age at which women and men reach peak fertility.

## METHODS

### Data

We test our hypotheses with 80 annual Swedish birth cohorts born from 1751 through 1830. Three circumstances make these cohorts particularly, if not uniquely, well suited for testing hypotheses such as ours. First, mothers of Swedish infants born in these years were themselves born in Sweden because emigration and immigration remained rare before 1830 [51]. Second, unlike most other substantial populations unperturbed by migration, vital statistics describing these Swedish birth cohorts meet minimum standards of accuracy and completeness for inclusion in the Human Mortality Database [28]. We know, for example, the mean peak age of fertility, approximately 32 years, among the mothers that produced these cohorts and can, therefore, identify the likely sex ratio of the mothers’ birth cohorts [52]. Third, these birth cohorts provide an opportunity to test hypothesis two—that an extreme sex ratio associated with an exogenous shock predicts opposite, though damped, outliers at a lag equal to the age at which women and men reach peak fertility. The Icelandic Laki volcano erupted from June 1783 through January 1784 and released an estimated 122 megatons of sulfur dioxide (SO_2_), hydrogen chloride and fluoride, and particulate matter into the atmosphere [31]. From Iceland, sulfate aerosol, 80 times greater than that produced in the Mount St. Helens eruption in 1980 traveled east, produced poor air quality and acid rain for several months, and withered crops after its late June arrival in Scandinavia [53, 54]. Research reports associations between the Laki event—likely driven by dramatically increased air pollution exposure [55] as well as reduced nutrition [56]—and reduced secondary sex ratios [31]. Indeed, Swedes produced their lowest recorded annual sex ratio (i.e. 1.021) in 1784. We, therefore, use that year to anchor our test of hypothesis two—that an extreme sex ratio associated with an exogenous shock should be followed by an opposite, though damped, outlier at a lag equal to that of the oscillation, if any, detected in the test of hypothesis one.

### Analyses

We test our first hypothesis, that deviations of the human secondary sex ratio around its mean exhibit damping oscillation over time, by estimating *q* and *θ* in the following equation.

(1) $$\left(m_{t}/f_{t}\right)=C+(1-\theta B^{q})e_{t}$$

Where

*m* is the count of Swedish males born in year *t*.

*f* is the count of Swedish females born in year *t*.

*C* is the mean of (*m*_t_/*f*_t_), or secondary sex ratios, for the test period.

*e* is the deviation from *C* of the secondary sex ratio at year *t*.

*θ* is a parameter that measures the fraction of *e* at year *t* − *q* which is ‘remembered’ and added to or subtracted from the secondary sex ratio at year *t*.

*B* is the ‘backshift operator’ or value of *e* at year *t* - *q*.

Support for Hamilton’s argument requires not only that *θ* differ significantly from 0, but also that *q* have a value ranging from 32 to 35 years. We derive this range from the assumption that mothers and fathers making the greatest contribution to the sex ratio of births in year *t* would be those born in years *t* − 32 to *t* − 35. We assume this for two reasons. First, very few births during our test period (i.e. 1751–830) occurred outside marriage and estimates of the average age of marriage vary around 25 years for women and 28 years for men [52]. Second, the average annual age of mothers at birth varied around 32 years as did age at female peak fertility [52]. If, therefore, the presumed tendency to produce offspring of one sex or the other were embedded mostly in women, we would expect a peak-to-trough oscillation in the sex ratio at about 32 years but closer to 35 years if embedded in men.

A large literature beginning with the work of Yule and continuing through the rule-setting work of Bartlett and Quenouille, to the integrative work of Box and Jenkins, Brillinger, and Royama has shown that the value of *q* can be estimated by comparing the autocorrelations and partial autocorrelations of a time series [32, 57–61]. Autocorrelations are essentially correlation coefficients between a variable, the annual Swedish secondary sex ratio in our test and its own earlier values such that the value at time *t* is predicted by values at time *t* − 1, *t* − 2 and so on. Partial autocorrelations are also essentially correlation coefficients between the secondary sex ratio and its earlier values, but the value at time *t* is predicted by values at time *t* − *n* adjusting for the association between the ratio and all its prior values before *t* − *n*. Autocorrelation coefficients are always bivariate whereas partial autocorrelation coefficients beyond *t* − 1 always ‘control’ for earlier associations.

Autocorrelations for oscillation show a significant coefficient at the peak-to-trough distance (i.e. *t* − *q*). If such a coefficient appears at the hypothesized time, at or near 32 years in our case, the partial autocorrelation function then provides information confirming oscillation rather than other forms of autocorrelation. The partial autocorrelations signaling oscillation shows not only significant autocorrelation at the peak-to-trough distance (i.e. at *t* − *q*), but also a negatively signed coefficient at twice that distance (i.e. *t* − 2*q*) that is absolutely larger than the autocorrelation coefficient at twice the peak-to-trough distance (i.e. *t* − 2*q*). If these conditions are met, we can specify *q* and estimate *θ*. Hamilton’s homeostasis implies, as noted above, that *q* for autocorrelations among Swedish annual sex ratios will range between 32 and 35.

We used two steps to test hypothesis two, that an extreme secondary sex ratio associated with an exogenous shock, should be followed by an opposite, though damped, outlier. First, we specified a Box–Jenkins transfer function by expanding Equation (1) to include a binary variable scored one for 1784 and zero otherwise to identify the birth cohorts exposed *in utero* to the Laki volcano [59]. Second, we estimated this transfer function using methods that detect outliers in time series adjusted for autocorrelation [62]. We set the definition of outlier as a year with sex ratio outside the 99% confidence interval after adjustment for endemic oscillation (i.e. autocorrelation) and the Laki event. If Hamilton’s logic describes the experience of the Swedish population during our test period, we should detect exogenously induced oscillation signaled by an outlying high ratio at or near 1816.

## RESULTS

Tables 1 and 2 show the autocorrelations and partial autocorrelations, and their Bartlett [57] standard errors for the sex ratio time series. The pattern of coefficients shows the signature of damping oscillation at 14 years rather than at 32–35 years, the age at which Swedish women and men reached peak fertility during this period, as predicted by Hamilton’s summary. Both the autocorrelation (i.e. −0.27, SE = 0.12) and partial autocorrelation (i.e. −0.25, SE = 0.11) coefficients at *t* − 14 fall below Bartlett’s 95% confidence interval. Also consistent with damping oscillation, the partial autocorrelation coefficient at *t* − 28 (i.e. −0.08) is absolutely larger than the autocorrelation coefficient at *t* − 28 (i.e. −0.03).

**Table 1.** Autocorrelations (Bartlett standard errors below) of the Swedish secondary sex ratio (1751 through 1830)

												
1–12	0.04	0.03	0.04	−0.12	−0.16	−0.06	−0.08	−0.03	0.12	0.11	0.06	0.06
ST.E.	0.11	0.11	0.11	0.11	0.11	0.12	0.12	0.12	0.12	0.12	0.12	0.12
13–24	0.03	−0.27*	−0.02	−0.10	−0.14	−0.01	0.04	0.12	0.05	0.10	−0.07	0.03
ST.E.	0.12	0.12	0.13	0.13	0.13	0.13	0.13	0.13	0.13	0.13	0.13	0.13
25–36	−0.11	−0.15	−0.13	−0.03	0.04	0.04	0.10	0.02	0.10	0.00	−0.04	−0.05
ST.E.	0.13	0.14	0.14	0.14	0.14	0.14	0.14	0.14	0.14	0.14	0.14	0.14

* *P* < 0.05, two-tailed test.

**Table 2.** Partial autocorrelations (Bartlett standard errors below) of the Swedish secondary sex ratio (1751 through 1830)

												
1–12	0.04	0.03	0.04	−0.12	−0.16	−0.05	−0.06	−0.02	0.10	0.08	0.02	0.01
ST.E.	0.11	0.11	0.11	0.11	0.11	0.11	0.11	0.11	0.11	0.11	0.11	0.11
13–24	0.03	−0.25*	0.02	−0.06	−0.08	−0.04	−0.02	0.09	−0.04	0.04	−0.07	0.08
ST.E.	0.11	0.11	0.11	0.11	0.11	0.11	0.11	0.11	0.11	0.11	0.11	0.11
25–36	−0.09	−0.10	−0.09	−0.08	0.06	−0.06	−0.00	−0.06	0.06	0.03	−0.04	0.05
ST.E.	0.11	0.11	0.11	0.11	0.11	0.11	0.11	0.11	0.11	0.11	0.11	0.11

* *P* < 0.05, two-tailed test.

Estimating Equation (1) with *q* set to 14 yielded the following values.

$$\left(m_{t}/f_{t}\right)=1.0457+\left(1-0.2984B^{14}\right)e_{t}$$

The estimates for *C* (i.e. 1.0457) and *θ* (i.e. 0.02984) both exceeded twice their standard errors (0.0006, *P* < 0.001, and 0.1128, *P* < 0.01, respectively).

In a population that should have exhibited damping oscillation from 32 to 35 years if Hamilton were correct, we found evidence that high or low values of the sex ratio triggered homeostasis that yielded damped symmetry 14 years later. If this 14-year oscillation were real, our second test would find exogenously induced oscillation in the form of an outlying sequence of high values starting at 1798 (i.e. 14 years after the Laki eruption). Outlier detection methods [62] found such a sequence that included significantly lower values than expected in 1798 and 1799.

The complete set of estimates for equation two appears as follows.

(2) $$\left(m_{t}/f_{t}\right)=1.0453-0.0273X_{1t}+0.0133/\left(1-0.7718B\right)X_{2t}+\left(1-0.3773B^{14}\right)e_{t}$$

Where

*X*_1t_ is a binary variable scored one for 1784 and zero otherwise.

*X*_2t_ is a binary variable scored one for 1798 and zero otherwise.

Other notation remains as described for Equation (1) above. All estimated coefficients exceeded twice their standard errors.

**Figure 1.**
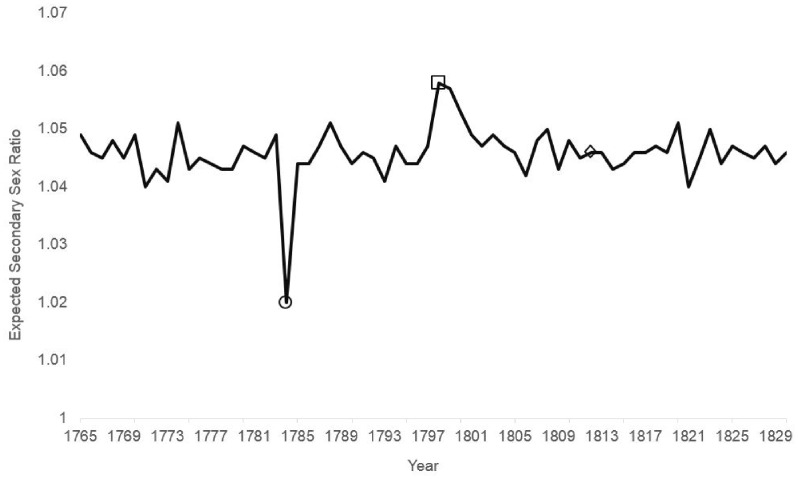
Estimated values produced by Box–Jenkins transfer function that models the Swedish secondary sex ratio from autocorrelation, the Laki volcano (shown with circle) and damping oscillation at 14 years (shown with square) and 28 (shown with diamond) years

Figure 1 shows the values estimated by Equation (2). The eye may not detect autocorrelation in which the value for each annual birth cohort predicts a smaller but opposite value 14 years later, but the superimposed oscillation of the Laki event appears detectable. The historic low ratio at 1784 ‘echoes’ inversely, but diminished, at 1798 and 1799 and declines again in 1812.

Estimating Equation (2)with the sex ratio transformed to natural logarithms allowed us to interpret the coefficients as percent changes in the odds of a male birth associated with the Laki event in 1784 and its ‘echo’. The odds of male birth dropped 2.6% in 1784, but oscillation ‘restored’ about 2.1% in 1798 and 1799.

## DISCUSSION

Sweden from 1751 through 1830 presents perhaps the best opportunity for testing oscillation in the human secondary sex ratio given little in- and out-migration, accurate vital statistics, a large number of births (mean = 71 886 annual births), and an extreme exogenous stressor (i.e. the Laki eruption). The relatively long time-series of sex ratios in Sweden allowed us, moreover, to use rigorous, sensitive and widely understood methods to detect and describe oscillation. If heritable mechanisms like those assumed by Düsing and Fisher affect the human secondary ratio, peak-to-trough oscillation at 32–35 years should appear in these data. We found oscillation but at 14 years. The finding implies that ‘compensating’ births had mothers with a mean age of 18 (i.e. 32 years less 14 = 18), or fathers with a mean age of 20, at the time outlying values in the secondary sex ratio appeared. Similarly, women and men at or around 18 or 20 years old respectively at the time of the Laki eruption likely accounted for the compensating high sex ratio in 1798.

We do not claim that Swedes living during our test period axiomatically represent all other humans. We note, however, that we know of no reason to believe that Swedes during the late eighteenth century somehow avoided whatever heritable mechanisms Fisher suspected and to which Hamilton alluded.

Our findings support the argument [41] that the human secondary sex ratio’s oscillation around 1 cannot arise from a heritable predisposition to produce males or females. Rather, mechanisms set in motion among persons approaching reproductive age at the time of the initial perturbation appear to contribute to compensatory oscillation. The literature describes plausible implications of the ‘adult’ sex ratio for mate selection and its sequelae but offers no explanation for why the ‘secondary’ sex ratio at the time of mate selection would predict the secondary sex ratio 14 years later [63].

Other literature not explicitly concerned with Düsing’s, Fisher’s or Hamilton’s arguments suggests mechanisms at least intuitively consistent with our finding of a 14-year distance between peak and trough in secondary sex ratios. Martin *et al.* [64], for example, argue that extreme adult sex ratios can affect the secondary sex ratio by shifting the age of peak sexual activity and childbearing among women seeking mates. The Laki event, for example may have caused women born in, or just before, the 1784 cohort to aggressively seek and secure mates at a relatively young age given the comparatively few males available. This circumstance would, supposedly, lead to relatively early and frequent coitus, as well as greater than expected fertility, all of which may contribute to high sex ratios [65]. We do not find this explanation of our findings compelling for three reasons. First, maternal age does not strongly predict sex of offspring [66]. Second, the age at mate selection and childbearing would have had to shift dramatically downward in 1798 but the few available data on age-specific fertility for that decade do not support such a shift [52]. And third, total births in 1798 did not exceed those expected from trend in years immediately before and after [28].

Other mechanisms described in the literature and intuitively consistent with our findings include the triggering of spontaneous abortion, particularly of small male fetuses, among gravid females witnessing aggression toward young conspecifics [67]. This ‘reproductive suppression’ supposedly reduces the chances that mothers will invest in sons likely to suffer disability or death due to inter-male aggression. Such selection *in utero* could account for our findings assuming adolescent males exhibit violence against competitors for female attention [68, 69], and that this violence varies positively with adolescent sex ratios [70, 71]. Under these assumptions, pregnant women embedded in a population with a low adolescent sex ratio might witness relatively less violence thereby reducing reproductive suppression and the spontaneous abortion of males. This argument, however, appears diminished by the fact that death among Swedish 10–14-year-old males in 1798 did not differ from trends shown in the years immediately before and after [28].

Hamilton’s widely cited, though rarely tested, summary of Düsing’s and Fisher’s arguments assumes that variation in the secondary sex ratio reflects inherited tendencies to produce males or females. We found no evidence of such tendencies in a human population particularly well suited to the test. The data suggest, rather, that other factors, such as cultural mechanisms dictating mating choices as well as natural selection *in utero*, instead determine the ratio of sons to daughters at birth.

Both historical and contemporary research finds that males born to high sex ratios show increased morbidity and mortality not only in childhood but also later in life [9]. Explanations for these associations invoke two general mechanisms. The first mechanism involves relaxed selection against frail males *in utero* such that a greater than expected fraction of these gestations—that is, those with a low likelihood of thriving once born—survive to birth [1]. Once born, males in this cohort face a greater average risk of morbidity and mortality over the life course. Literature from diverse fields identifies several markers of fetal fitness (e.g. chromosomal anomaly, growth restriction, hormone levels predictive of spontaneous abortion) that vary with the secondary sex ratio and appear consistent with this selection *in utero* mechanism [19].

The second mechanism involves preferential parental investment in healthier children once born. According to life-history theory, a mother constantly makes investment tradeoffs not only during, but also after, pregnancy [72, 73]. These post-pregnancy tradeoffs include, for example, (i) childrearing effort versus maintenance of nonreproductive biology; (ii) current children versus future reproductive effort; and (iii) differential childrearing effort across children. According to the theory, mothers may invest relatively more childrearing effort in children deemed most likely to thrive and yield her grandchildren. Contemporary data support this differential investment mechanism. In the USA, mothers with several children show a greater likelihood of investing (e.g. breast-feeding, well-baby visits, immunizations) in children born at a normal (vs lower) birthweight [74]. In a separate study, parents with two children eligible for a beneficial educational program chose to enroll the child with better health at birth [75]. This work supports the notion that post-reproductive parental investments tend to reinforce, over the life course, within-child differences in health endowments at birth. We know of no literature that examines whether males born to high or low sex ratio cohorts undergo relatively higher or lower levels of parental investment over the life course. We recommend empirical studies on this topic given extensive research documenting the importance of early childhood investments on human and health capital into adulthood [76–78].

Explaining variation over time in the human secondary sex ratio would seem important for public health for two reasons. First, such changes may provide opportunities to understand gestation and fetal loss. Unexpectedly low sex ratios may serve an important surveillance function given that most literature documents elevated selection *in utero* among male fetuses in response to environmental perturbations [4]. Second, sex ratios predict the health and longevity of populations [1–9]. This circumstance indicates that public health efforts designed to focus on subgroups at elevated risk of disease and death may consider monitoring males born to cohorts with unusually high sex ratios. Such monitoring may also include empirical studies, within a life-history theory framework, that examine potential causes of, and differential parental investment in, children born to unusually high sex ratio cohorts.

Conflict of interest: None declared.

## References

[1] Trivers RL, Willard DE. Natural selection of parental ability to vary the sex ratio of offspring. Science 1973;179:90–2. 4682135 10.1126/science.179.4068.90

[2] James WH, Grech V. Can sex ratios at birth be used in the assessment of public health, and in the identification of causes of selected pathologies? Early Hum Dev 2018;118:15–21. 29428574 10.1016/j.earlhumdev.2018.02.003

[3] Catalano R, Bruckner T. Secondary sex ratios and male lifespan: damaged or culled cohorts. Proc Natl Acad Sci USA 2006;103:1639–43. 16432236 10.1073/pnas.0510567103 PMC1360590

[4] Bruckner TA, Helle S, Bolund E et al Culled males, infant mortality and reproductive success in a pre-industrial Finnish population. Proc R Soc B Biol Sci 2015;282:20140835. 10.1098/rspb.2014.0835 PMC4286058 25621334

[5] Bruckner TA, Nobles J. Intrauterine stress and male cohort quality: the case of September 11, 2001. Soc Sci Med 2013;76:107–14. 23153542 10.1016/j.socscimed.2012.10.012

[6] Goodman JM, Karasek D, Anderson E et al The contribution of attenuated selection in utero to small-for-gestational-age (SGA) among term African American male infants. Soc Sci Med 2013;88:83–9. 23702213 10.1016/j.socscimed.2013.04.006

[7] Singh P, Yang W, Shaw GM et al Selected birth defects among males following the United States terrorist attacks of 11 September 2001. Birth Defects Res 2017;109:1277–83. 28722355 10.1002/bdr2.1072

[8] Bruckner TA, Karasek D, Yang W et al Cohort variation in selection during pregnancy and risk of selected birth defects among males. Epidemiology 2017;28:580–6. 28346269 10.1097/EDE.0000000000000661

[9] Bruckner T, Catalano R. The sex ratio and age-specific male mortality: evidence for culling in utero. Am J Hum Biol 2007;19:763–73. 17676612 10.1002/ajhb.20636

[10] Düsing C. Die Regulierung des Geschlechtsverha ltnisses bei der Vermehrung der Menschen, Tiere und Pflanzen. Jenaische Zeitschrift Für Naturwissenschaft 1884;17:593–940.

[11] Edwards AW. Carl Düsing (1884) on the regulation of the sex-ratio. Theor Popul Biol 2000;58:255–7. 11120652 10.1006/tpbi.2000.1482

[12] Fisher RA. The Genetical Theory of Natural Selection. Oxford: Oxford University Press, 1930.

[13] Hamilton WD. Extraordinary sex ratios. Science 1967;156:477–88. 6021675 10.1126/science.156.3774.477

[14] Torche F, Kleinhaus K. Prenatal stress, gestational age and secondary sex ratio: the sex-specific effects of exposure to a natural disaster in early pregnancy. Hum Reprod 2012;27:558–67. 22157912 10.1093/humrep/der390 PMC3258031

[15] Catalano RA. Sex ratios in the two Germanies: a test of the economic stress hypothesis. Hum Reprod 2003;18:1972–5. 12923159 10.1093/humrep/deg370

[16] Catalano R, Bruckner T, Smith KR. Ambient temperature predicts sex ratios and male longevity. Proc Natl Acad Sci USA 2008;105:2244–7. 18250336 10.1073/pnas.0710711104 PMC2538905

[17] James WH. Hypotheses on the stability and variation of human sex ratios at birth. J Theor Biol 2012;310:183–6. 22776504 10.1016/j.jtbi.2012.06.038

[18] James WH. Proximate causes of the variation of the human sex ratio at birth. Early Hum Dev 2015;91:795–9. 26549774 10.1016/j.earlhumdev.2015.10.004

[19] Bruckner TA, Catalano R. Selection in utero and population health: theory and typology of research. Soc Sci Med-Population Health 2018;5:101–13. 10.1016/j.ssmph.2018.05.010 PMC6008283 29928686

[20] Boklage CE. Survival probability of human conceptions from fertilization to term. Int J Fertil 1990;35:75–94. 1970983

[21] Orzack SH, Stubblefield JW, Akmaev VR et al The human sex ratio from conception to birth. Proc Natl Acad Sci USA 2015;112:E2102–11. 25825766 10.1073/pnas.1416546112 PMC4413259

[22] McKeown T, Lowe CR. Sex ratio of stillbirths related to birth weight. Br J Soc Med 1951;5:229–35. 14886590 10.1136/jech.5.4.229 PMC1037299

[23] Mondal D, Galloway TS, Bailey TC et al Elevated risk of stillbirth in males: systematic review and meta-analysis of more than 30 million births. BMC Med 2014;12:220. 25428603 10.1186/s12916-014-0220-4 PMC4245790

[24] Räisänen S, Gissler M, Saari J et al Contribution of risk factors to extremely, very and moderately preterm births–register-based analysis of 1,390,742 singleton births. PLoS One 2013;8:e60660. 23577142 10.1371/journal.pone.0060660 PMC3618176

[25] Quenby S, Vince G, Farquharson R et al Recurrent miscarriage: a defect in nature’s quality control? Hum Reprod 2002;17:1959–63. 12151421 10.1093/humrep/17.8.1959

[26] Lummaa V, Jokela J, Haukioja E. Gender difference in benefits of twinning in pre‐industrial humans: boys did not pay. J Anim Ecol 2001;70:739–46.

[27] Lummaa V. Reproductive investment in pre-industrial humans: the consequences of offspring number, gender and survival. Proc R Soc B Biol Sci 2001;268:1977–83. 10.1098/rspb.2001.1786 PMC1088838 11571043

[28] Human Mortality Database. University of California, Berkeley (USA), and Max Planck Institute for Demographic Research (Germany). www.mortality.org or www.humanmortality.de, (16 November 2016, date last accessed).

[29] Catalano R, Bruckner T, Marks AR et al Exogenous shocks to the human sex ratio: the case of September 11, 2001 in New York City. Hum Reprod 2006;21:3127–31. 16936298 10.1093/humrep/del283

[30] Catalano RA, Saxton KB, Gemmill A et al Twinning in Norway following the Oslo Massacre: evidence of a ‘Bruce effect’ in humans. Twin Res Hum Genet 2016;19:485–91. 27453297 10.1017/thg.2016.58

[31] Casey JA, Gemmill A, Elser H et al Sun smoke in Sweden: perinatal implications of the Laki volcanic eruptions, 1783–1784. Epidemiology 2019;30:330–3. 30789427 10.1097/EDE.0000000000000977 PMC6456407

[32] Yule G. On a method of investigating periodicities in disturbed series, with special reference to Wolfer’s sunspot numbers. Philos T R Soc A 1927;226:267–98.

[33] Frank SA. Hierarchical selection theory and sex ratios I. General solutions for structured populations. Theor Popul Biol 1986;29:312–42. 3738836 10.1016/0040-5809(86)90013-4

[34] Cockburn A, Legge S, Double MC. Sex ratios in birds and mammals: can the hypotheses be disentangled In: Hardy I (ed.). Sex Ratios: Concepts and Research Methods. Cambridge, MA: Cambridge University Press, 2002, 266–86.

[35] Bernstein ME. Action of genes affecting secondary sex ratio in man. Science 1951;114:181–2. 10.1126/science.114.2955.181-a 14866183

[36] Edwards A. Genetics and the human sex ratio. Adv Genet 1962;11:239–72.

[37] Curtsinger JW, Ito R, Hiraizumi Y. A two-generation study of human sex-ratio variation. Am J Hum Genet 1983;35:951–61. 6614009 PMC1685814

[38] Astolfi P, Cuccia M, Martinetti M. Paternal HLA genotype and offspring sex ratio. Hum Biol 2001;73:315–9. 11446432 10.1353/hub.2001.0015

[39] Rodgers JL, Doughty D. Does having boys or girls run in the family? Chance 2001;14:8–13.

[40] Gellatly C. Trends in population sex ratios may be explained by changes in the frequencies of polymorphic alleles of a sex ratio gene. Evol Biol 2009;36:190–200.

[41] Zietsch BP, Walum H, Lichtenstein P et al No genetic contribution to variation in human offspring sex ratio: a total population study of 4.7 million births. Proc R Soc B Biol Sci 2020;287:20192849. 10.1098/rspb.2019.2849 PMC7062014 32070249

[42] Graffelman J, Hoekstra RF. A statistical analysis of the effect of warfare on the human secondary sex ratio. Hum Biol 2000;72:433–45. 10885189

[43] Lummaa V, Merilä J, Kause A. Adaptive sex ratio variation in pre-industrial human (Homo sapiens) populations? Proc Biol Sci 1998;265:563–8. 9881467 10.1098/rspb.1998.0331 PMC1689011

[44] Ranta E, Lummaa V, Kaitala V et al Spatial dynamics of adaptive sex ratios. Ecol Lett 2000;3:30–4.

[45] Polasek O. Did the 1991–1995 wars in the former Yugoslavia affect sex ratio at birth? Eur J Epidemiol 2006;21:61–4. 16450208 10.1007/s10654-005-4845-7

[46] James WH. Secular movements in sex ratios of adults and of births in populations during the past half-century. Hum Reprod 2000;15:1178–83. 10783374 10.1093/humrep/15.5.1178

[47] Helle S, Käär P, Helama S, Jokela J. Do humans adjust offspring sex according to local operational sex ratio? Evol Ecol Res 2008;10:775–85.

[48] Song S. Evidence of adaptive intergenerational sex ratio adjustment in contemporary human populations. Theor Popul Biol 2014;92:14–21. 24240059 10.1016/j.tpb.2013.10.006

[49] Belsky J, Steinberg L, Draper P. Childhood experience, interpersonal development, and reproductive strategy: an evolutionary theory of socialization. Child Dev 1991;62:647–70. 1935336 10.1111/j.1467-8624.1991.tb01558.x

[50] Almond D, Li H, Zhang S. Land reform and sex selection in China. J Polit Econ 2019;127:560–85.

[51] Statistics Sweden. Population Development in Sweden in a 250-Year Perspective. Stockholm: Statistics Sweden, 1999.

[52] National Central Bureau of Statistics. Historical Statistics of Sweden. Part 1. Population, Second Edition, 1720–1967. Stockholm: National Central Bureau of Statistics, 1969.

[53] Decker R, Decker B. The eruptions of Mount St. Helens. Sci Am 1981;244:68–81.

[54] Thordarson T, Self S. Atmospheric and environmental effects of the 1783–1784 Laki eruption: a review and reassessment. J Geophys Res Atmos 2003;108:AAC-7.

[55] Lichtenfels A, Gomes J, Pieri P et al Increased levels of air pollution and a decrease in the human and mouse male-to-female ratio in São Paulo, Brazil. Fertil Steril 2007;87:230–2. 17084397 10.1016/j.fertnstert.2006.06.023

[56] Song S. Does famine influence sex ratio at birth? Evidence from the 1959–1961 Great Leap Forward Famine in China. Proc R Soc B Biol Sci 2012;279:2883–90. 10.1098/rspb.2012.0320 PMC3367790 22456881

[57] Bartlett MS. On the theoretical specification and sampling properties of autocorrelated time-series. J R Stat Soc 1946;8:27–41.

[58] Quenouille MH. Approximate tests of correlation in time-series 3. Math Proc Camb Philos Soc 1949;45:483–4.

[59] Box G, Jenkins G. Time Series Analysis: Forecasting and Control. San Francisco, CA: Holden-Day, 1976.

[60] Brillinger D. Time Series: Data Analysis and Theory. San Francisco, CA: Holden‐Day, 1981.

[61] Royama T. Analytical Population Dynamics. London: Chapman & Hall, 1992.

[62] Chang I, Tiao GC, Chen C. Estimation of time series parameters in the presence of outliers. Technometrics 1988;30:193–204.

[63] Jennions MD, Fromhage L. Not all sex ratios are equal: the Fisher condition, parental care and sexual selection. Philos T R Soc B 2017;372:20160312. 10.1098/rstb.2016.0312 PMC5540854 28760755

[64] Martin JF, Hammel EA, Harris M et al Changing sex ratios: the history of havasupai fertility and its implications for human sex ratio variation. Curr Anthropol 1994;35:255–80.

[65] James WH. The honeymoon effect on marital coitus. J Sex Res 1981;17:114–23.

[66] Rueness J, Vatten L, Eskild A. The human sex ratio: effects of maternal age. Hum Reprod 2012;27:283–7. 22025225 10.1093/humrep/der347

[67] Saxton KB, Gemmill A, Catalano RA. Reproductive suppression follows threats to child survival. J Evol Biol 2017;30:889–97. 28267227 10.1111/jeb.13061

[68] Borowsky IW, Hogan M, Ireland M. Adolescent sexual aggression: risk and protective factors. Pediatrics 1997;100:e7. 10.1542/peds.100.6.e7 9382908

[69] Volk AA, Camilleri JA, Dane AV, Marini ZA. Is adolescent bullying an evolutionary adaptation? Aggress Behav 2012;38:222–38. 22331629 10.1002/ab.21418

[70] Diamond-Smith N, Rudolph K. The association between uneven sex ratios and violence: evidence from 6 Asian countries. PLoS One 2018;13:e0197516. 29856763 10.1371/journal.pone.0197516 PMC5983495

[71] Campbell A. A few good men: evolutionary psychology and female adolescent aggression. Ethol Sociobiol 1995;16:99–123.

[72] Vitzthum V. Evolutionary models of women’s reproductive functioning. Annu Rev Anthropol 2008;37:53–73.

[73] Wells JCK, Nesse RM, Sear R et al Evolutionary public health: introducing the concept. Lancet 2017;390:500–9. 28792412 10.1016/S0140-6736(17)30572-X

[74] Datar A, Kilburn MR, Loughran DS. Endowments and parental investments in infancy and early childhood. Demography 2010;47:145–62. 20355688 10.1353/dem.0.0092 PMC3000015

[75] Aizer A, Cunha F. The production of human capital: endowments, investments and fertility. National Bureau of Economic Research Working Paper No. 18429, 2012.

[76] Campbell F, Conti G, Heckman JJ et al Early childhood investments substantially boost adult health. Science 2014;343:1478–85. 24675955 10.1126/science.1248429 PMC4028126

[77] Schoellman T. Early childhood human capital and development. Am Econ J Macroecon 2016;8:145–74.

[78] Gluckman PD, Hanson MA, Cooper C, Thornburg KL. Effect of in utero and early-life conditions on adult health and disease. N Engl J Med 2008;359:61–73. 18596274 10.1056/NEJMra0708473 PMC3923653

